# Fibrostenotic intestinal stenosis associated with chronic enteropathy in two cats: case report

**DOI:** 10.3389/fvets.2026.1950860

**Published:** 2026-10-12

**Authors:** Jiho Choi, Seongmo Yang, Yelim Lee, Yunjin Sung, Yeon Chae, Taesik Yun, Byeong-Teck Kang, Soochong Kim, Sungin Lee, Hakhyun Kim

**Affiliations:** 1 Laboratory of Veterinary Internal Medicine, College of Veterinary Medicine, Chungbuk National University, Cheongju, Chungbuk, Republic of Korea; 2 Laboratory of Veterinary Pathology and Platelet Signaling, Chungbuk National University, Cheongju, Chungbuk, Republic of Korea; 3 Department of Veterinary Surgery, College of Veterinary Medicine, Chungbuk National University, Cheongju, Chungbuk, Republic of Korea

**Keywords:** feline, intestinal fibrosis, JAK-STAT signaling, lymphoma, lymphoplasmacytic enteritis

## Abstract

Two cats presented with acute progressive vomiting. One cat (Case 1) received prednisolone for suspected chronic inflammatory enteropathy for 14 months. The cat showed hypoalbuminemia, hypocobalaminemia, small intestinal thickening, and focal jejunal stricture with severe proximal dilation on ultrasonography. Exploratory laparotomy confirmed the stricture, and histopathology revealed submucosal fibroplasia with mild lymphoplasmacytic enteritis. The cat died on postoperative day 12. Another cat (Case 2) showed proximal small intestinal obstruction on ultrasonography. A wide-margin resection of the obstructed lesion was performed. Histopathology revealed mild intestinal wall fibrosis with mesenteric inflammation. Masson’s trichrome staining showed submucosal collagen bundles. Immunohistochemistry and clonality analysis by PCR for antigen receptor rearrangement supported the diagnosis of low-grade intestinal T-cell lymphoma. The cat recovered uneventfully and remained restenosis-free during prednisolone treatment for 12 months after surgery. Intestinal stenoses were identified during long-term medical management of chronic enteropathy. This suggests that the extent of the lesion and the surgical resection strategy could be relevant factors for management and prognosis.

## 1 Introduction

Chronic enteropathy (CE) in cats encompasses various disorders of the small intestine, including lymphoplasmacytic enteritis (LPE) and low-grade intestinal T-cell lymphoma (LGITL), which are common entities that are difficult to distinguish clinically and histologically (1–4). Although LPE and LGITL have traditionally been regarded as inflammatory and neoplastic disorders, respectively, they can overlap in histologic features, immunophenotypes, and molecular findings. Thus, differentiation remains challenging even with WSAVA histopathological criteria, immunohistochemistry, and clonality testing (1–3, 5–7). These observations support the existence of a possible biological spectrum with overlapping features between inflammatory CE and LGITL (1–4).

In humans, intestinal fibrosis is a recognized feature of chronic inflammatory bowel disease, particularly Crohn’s disease; however, fibrotic remodeling does not necessarily progress to clinically relevant stricture formation (8, 9). In cats, fibrotic small intestinal strictures causing clinically relevant luminal narrowing or obstruction have been reported less frequently (10). Recent studies have identified intestinal fibrosis as a frequent histopathological finding in cats with chronic inflammatory enteropathy, and fibrosis may be associated with unfavorable clinical outcomes (11, 12). However, progression to clinically relevant fibrotic small intestinal strictures remains poorly documented, and longitudinal observation of this process during medical management is limited (10–12).

Persistent intestinal inflammation may contribute to fibrotic mural remodeling, but mechanisms in cats remain unclear. STAT3-related signaling is implicated in chronic intestinal inflammation in human inflammatory bowel disease (13). In feline LPE and LGITL, evaluation of STAT3 immunoreactivity may provide exploratory information regarding its potential involvement in intestinal wall remodeling.

This report describes two cats within the CE spectrum, one with LPE and one with LGITL, each of which developed a fibrotic small intestinal stricture during long-term medical management. We further describe histochemical, immunohistochemical, molecular, and JAK-STAT-related findings to explore the possible relationship among chronic intestinal inflammation, fibrotic remodeling, and stricture formation in these cases.

## 2 Case description

### 2.1 Case 1

An 11-year-old spayed female Norwegian Forest cat was referred for chronic vomiting. Physical examination revealed body weight of 3.69 kg, body condition score of 4/9, and estimated 7% dehydration. Serum biochemical analysis revealed hypoalbuminemia [1.7 g/dL; reference interval (RI), 2.1–3.3 g/dL], hyperglobulinemia (5.9 g/dL; RI, 2.6–5.1 g/dL), and decreased serum cobalamin concentration (198 pg/mL; RI, 276–1,425 pg/mL); oral cobalamin was initiated. Abdominal ultrasonography revealed diffuse small intestinal wall thickening, most prominently at the ileocecocolic junction (Figures 1A,B). CE was suspected. Initial treatment included dietary modification and supportive medical management. As the clinical response was incomplete, prednisolone (1 mg/kg PO every 12 h) was initiated and the clinical response to treatment was monitored.

**Figure 1:**
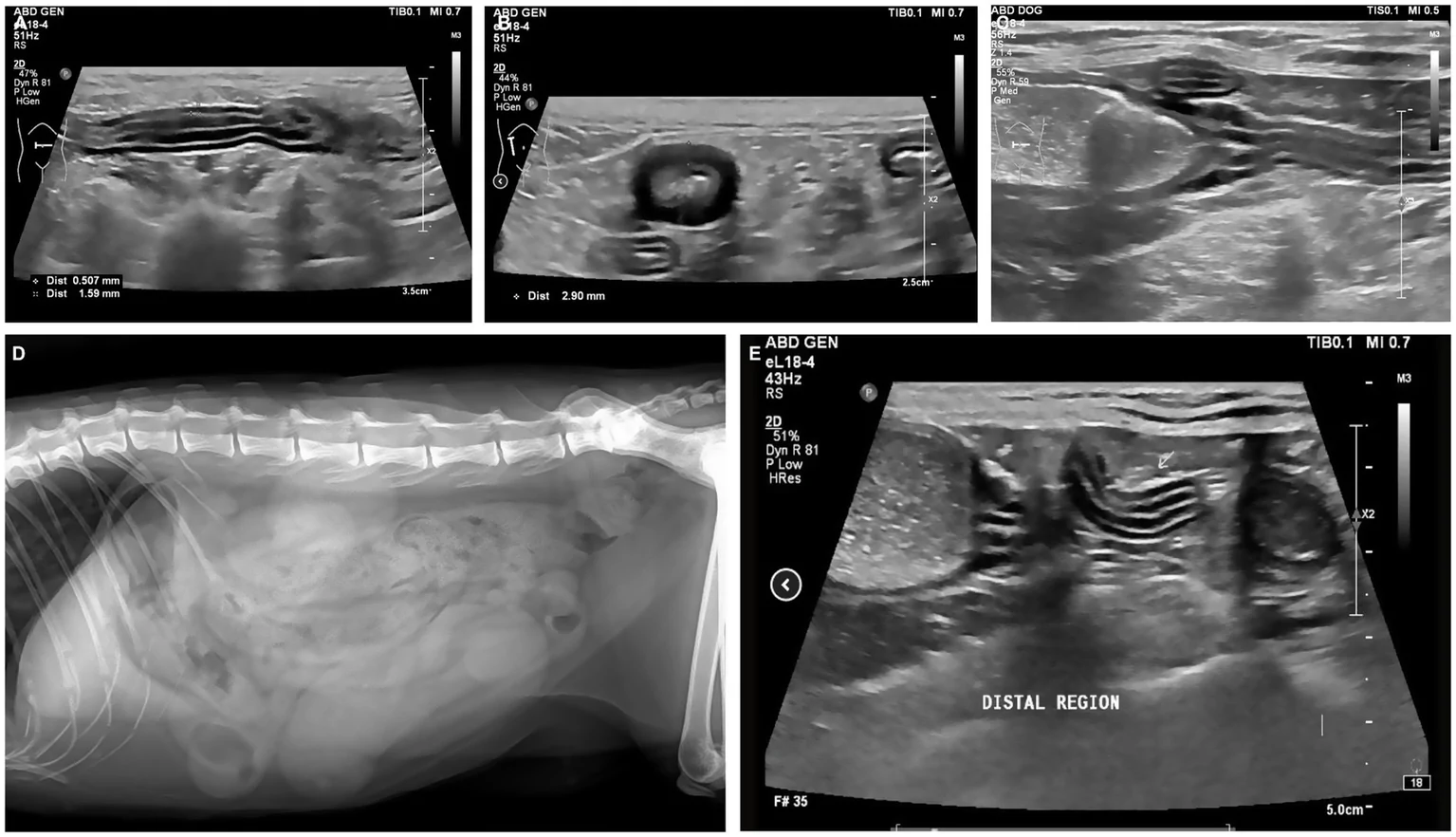
Ultrasound and radiographic findings of the fibrostenotic lesion in Case 1. **(A,B)** Abdominal ultrasonographic images obtained at initial evaluation showing diffuse small intestinal wall thickening, with prominent muscularis layer thickening at the ileocecocolic junction. **(C)** Follow-up ultrasonographic image obtained 421 days after initiation of medical management, showing a newly developed focal midjejunal stenotic lesion with proximal small intestinal dilation. **(D,E)** Right lateral abdominal radiographic and ultrasonographic images obtained 423 days after initiation of medical management, showing marked proximal small intestinal dilation and mechanical obstruction associated with the stenotic jejunal segment. The arrow in panel **E** indicates the narrowed intestinal lumen.

Over the following months, serum albumin increased, ileal wall thickness decreased, and vomiting resolved. Prednisolone was tapered, and the cat remained clinically stable without substantial ultrasonographic progression for approximately 14 months.

After 421 days of treatment, the cat developed recurrent vomiting, weight loss, and hypoalbuminemia (1.8 g/dL; RI, 2.1–3.3 g/dL). A repeat abdominal ultrasonography revealed a new midjejunal stenotic lesion with proximal small intestinal dilation (Figure 1C). Two days later, acute anorexia developed, and radiography and ultrasonography confirmed mechanical small intestinal obstruction (Figures 1D,E). Exploratory laparotomy, segmental resection, and end-to-end jejunojejunal anastomosis were performed. Histopathology revealed LPE with a segmental fibrostenotic lesion, submucosal fibroplasia, mature granulation tissue, and marked submucosal fibrosis (Figure 2A). Masson’s trichrome staining revealed submucosal collagen deposition (Figure 2C). Immunohistochemistry showed predominantly CD3-positive T lymphocytes and few PAX5-positive B cells (Figures 2E,G). Additional JAK-STAT-related immunohistochemistry was performed for total STAT3, total STAT5b, and phospho-JAK1. Details of the primary antibodies and controls are provided in Supplementary Table 1. Immunolabeling was evaluated semi-quantitatively in approximately five representative microscopic fields for each evaluated cell population. The proportion of immunoreactive cells was visually estimated, and staining intensity was categorized as weak, moderate, or strong. Total STAT3 staining was strongly multifocal in lamina propria lymphocytes, diffusely moderate in enterocytes, and diffusely strong in submucosal lymphocytes and fibroblasts (Figures 3A,C). Total STAT5b showed strong multifocal expression in lamina propria lymphocytes, diffuse weak staining in enterocytes, and scattered positivity in submucosal lymphocytes (Figure 3E). Phospho-JAK1 displayed strong multifocal staining in lamina propria lymphocytes and predominantly weak cytoplasmic immunoreactivity in enterocytes, with scattered strong staining in a small proportion of cells (Figure 3G). The semi-quantitative immunohistochemical findings are summarized in Supplementary Table 2.

**Figure 2:**
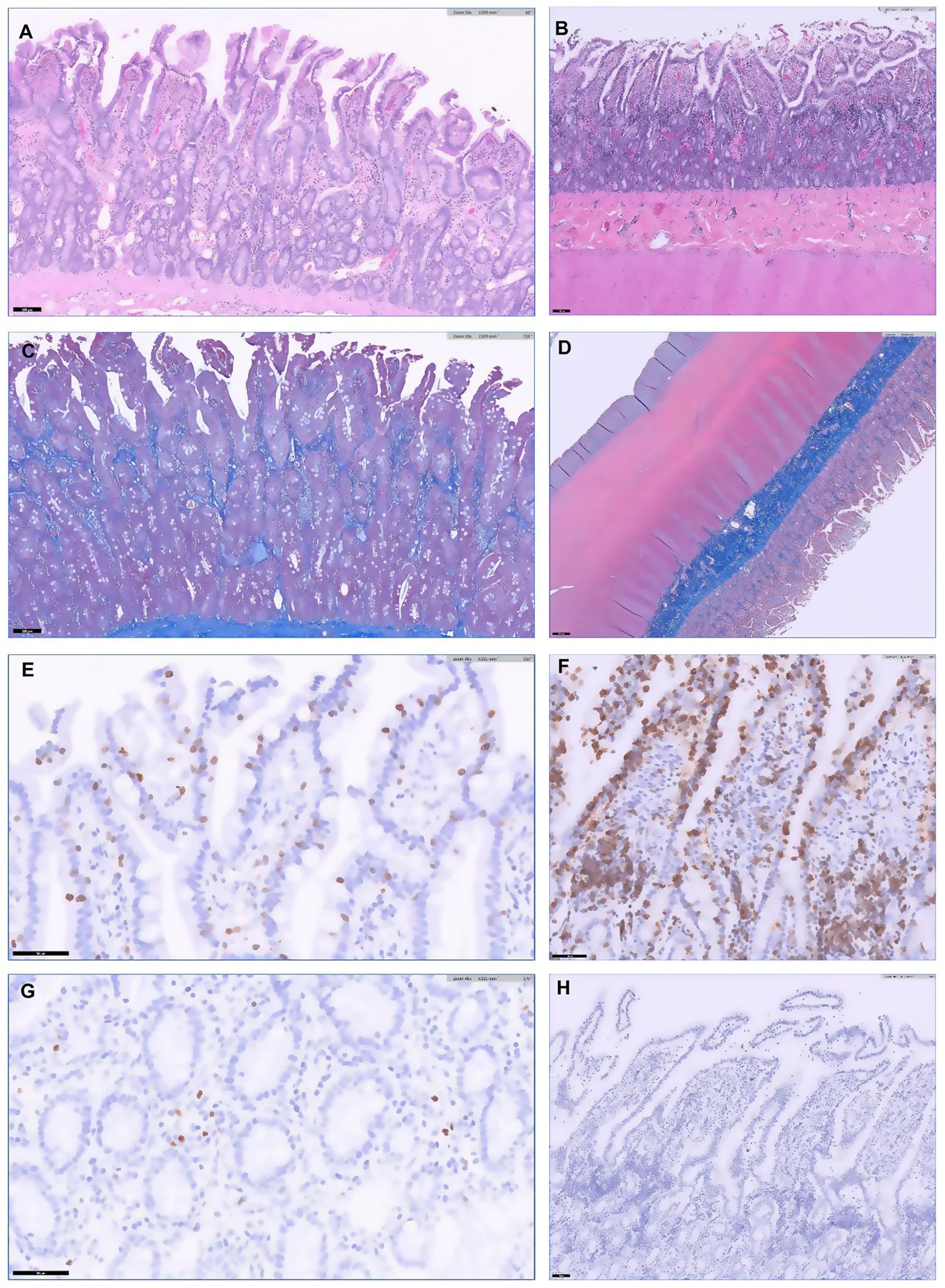
Histopathologic and immunophenotypic findings of the fibrostenotic lesions in the present cases. **(A,B)** H&E-stained sections of the resected jejunum show lymphoplasmacytic enteritis with submucosal fibroplasia, mature granulation tissue, and marked submucosal fibrosis in Case 1, whereas Case 2 shows fibroinflammatory enteritis with dense mucosal lymphocytic infiltration and mural remodeling. **(C,D)** Masson’s trichrome-stained sections show extensive submucosal collagen deposition in Case 1, whereas Case 2 shows submucosal collagen bundles and mild fibrosis within the overlying mucosa. **(E,F)** CD3 immunohistochemistry showing predominantly T-cell infiltration in both cases. **(G,H)** PAX5 immunohistochemistry showing few B lymphocytes in Case 1 and no detectable B-cell infiltrates in Case 2. Scale bars = 100 μm **(A,C,H)**, 200 μm **(B)**, 500 μm **(D)**, and 50 μm **(E–G)**.

**Figure 3:**
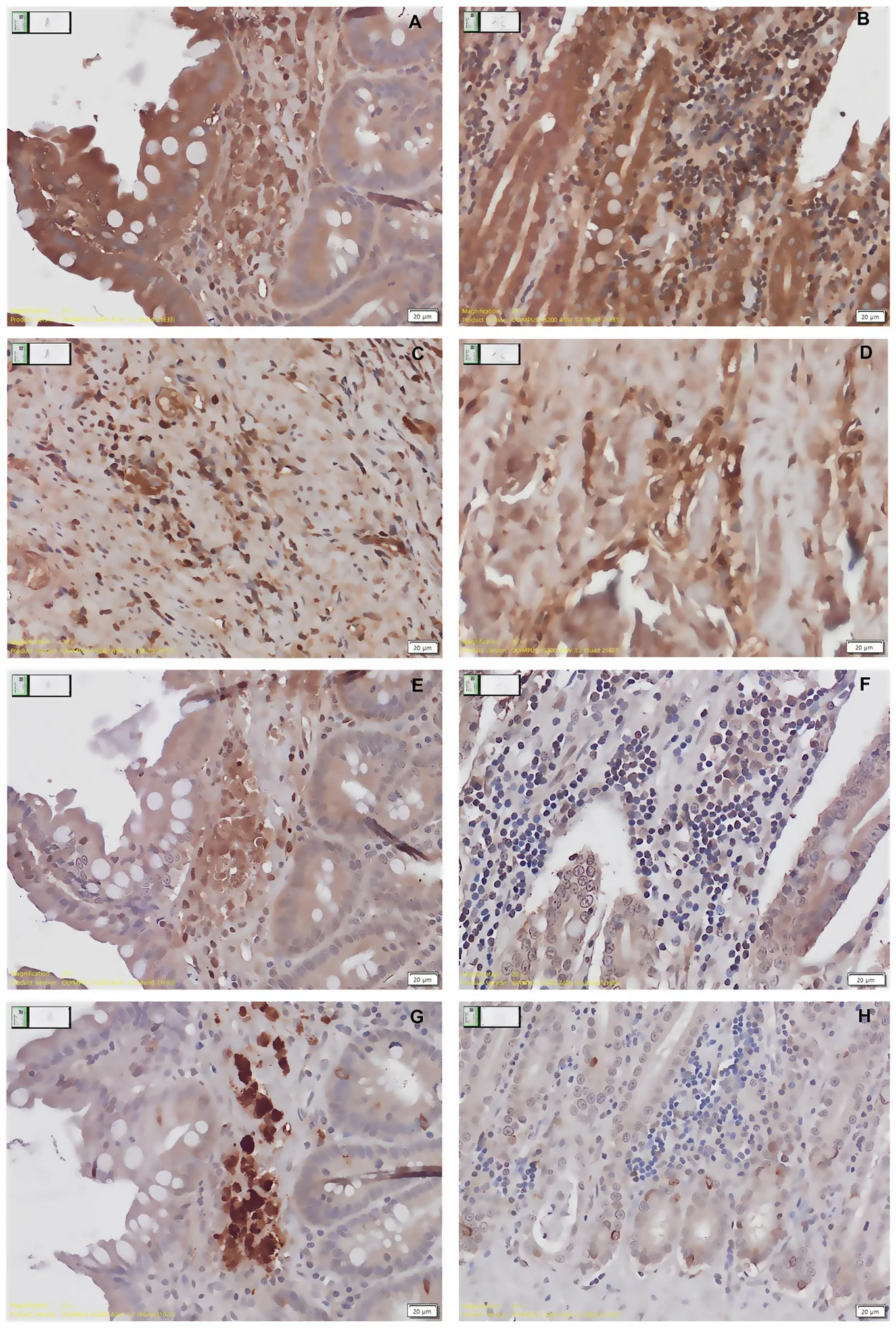
JAK-STAT-related immunohistochemical findings in Cases 1 and 2. **(A,B)** Total STAT3 immunoreactivity in the intestinal mucosa shows multifocal strong cytoplasmic immunoreactivity in lamina propria lymphocytes and diffuse moderate immunoreactivity in enterocytes of Case 1, and diffuse cytoplasmic immunoreactivity in a moderate proportion of mucosal lymphocytes and diffuse strong immunoreactivity in enterocytes of Case 2. **(C,D)** Total STAT3 immunoreactivity in the intestinal submucosa shows strong immunoreactivity in submucosal lymphocytes and fibroblasts in Case 1, whereas macrophages, neutrophils, and fibroblasts show diffuse immunoreactivity in Case 2. **(E,F)** Total STAT5b immunoreactivity in the intestinal mucosa shows multifocal strong immunoreactivity in lamina propria lymphocytes and diffuse weak immunoreactivity in enterocytes of Case 1, and rare immunoreactivity in mucosal lymphocytes and diffuse weak immunoreactivity in enterocytes of Case 2, respectively. **(G,H)** Phospho-JAK1 immunoreactivity in the intestinal mucosa shows multifocal strong immunoreactivity in lamina propria lymphocytes and predominantly weak cytoplasmic immunoreactivity in enterocytes, with scattered strong staining in a small proportion of cells, in Case 1 **(G)**. In Case 2 **(H)**, enterocytes show predominantly weak cytoplasmic immunoreactivity with scattered strong staining in a small proportion of cells. Scale bars = 20 μm.

Postoperative recovery was initially uneventful, and appetite and fecal quality normalized after discharge. However, the cat died suddenly at home on postoperative day 12.

### 2.2 Case 2

A 13-year-old neutered male domestic shorthair cat was referred for the evaluation of a 2-month history of chronic vomiting and progressive weight loss. On presentation, clinicopathologic abnormalities included anemia, indicated by a decreased packed cell volume (19.8%; RI, 30.3–52.3%), hypoalbuminemia (1.3 g/dL; RI, 2.1–3.3 g/dL), and a marked increase in serum amyloid A concentration (430 μg/mL; RI, 0–10 μg/mL). Abdominal radiography revealed severe segmental small-intestinal dilation (Figure 4A). Ultrasonography revealed an abrupt stenosis of the descending duodenum with proximal dilation, diffuse muscularis thickening, and mild mesenteric lymphadenopathy (Figures 4B,C). Contrast-enhanced computed tomography (CT) revealed a focal jejunal stenotic lesion causing mechanical obstruction (Figures 4D,E). Exploratory laparotomy identified a markedly inflamed jejunal segment containing the stenotic region, and wide-margin resection including the adjacent grossly normal bowel was performed, followed by end-to-end anastomosis. Histopathological examination of the resected jejunal segment revealed fibro-inflammatory enteritis with fibrosis and secondary stricture formation (Figure 2B). Masson’s trichrome staining revealed collagen deposition (Figure 2D). Immunohistochemistry demonstrated predominantly CD3-positive T-cell infiltration with no detectable PAX5-positive B-cell infiltrates (Figures 2F,H). Polymerase chain reaction for antigen receptor rearrangement (PARR) was performed (VDx Veterinary Diagnostics, Davis, CA, USA) using DNA extracted from formalin-fixed, paraffin-embedded jejunal tissue. Published revised primers targeting feline T-cell receptor gamma (TRG) and immunoglobulin heavy-chain framework 2 and 3 regions were used, and the amplicons were analyzed by capillary electrophoresis (14, 15). A clonal TRG pattern was identified, which, together with the histopathologic and immunophenotypic findings, supported the diagnosis of LGITL.

**Figure 4:**
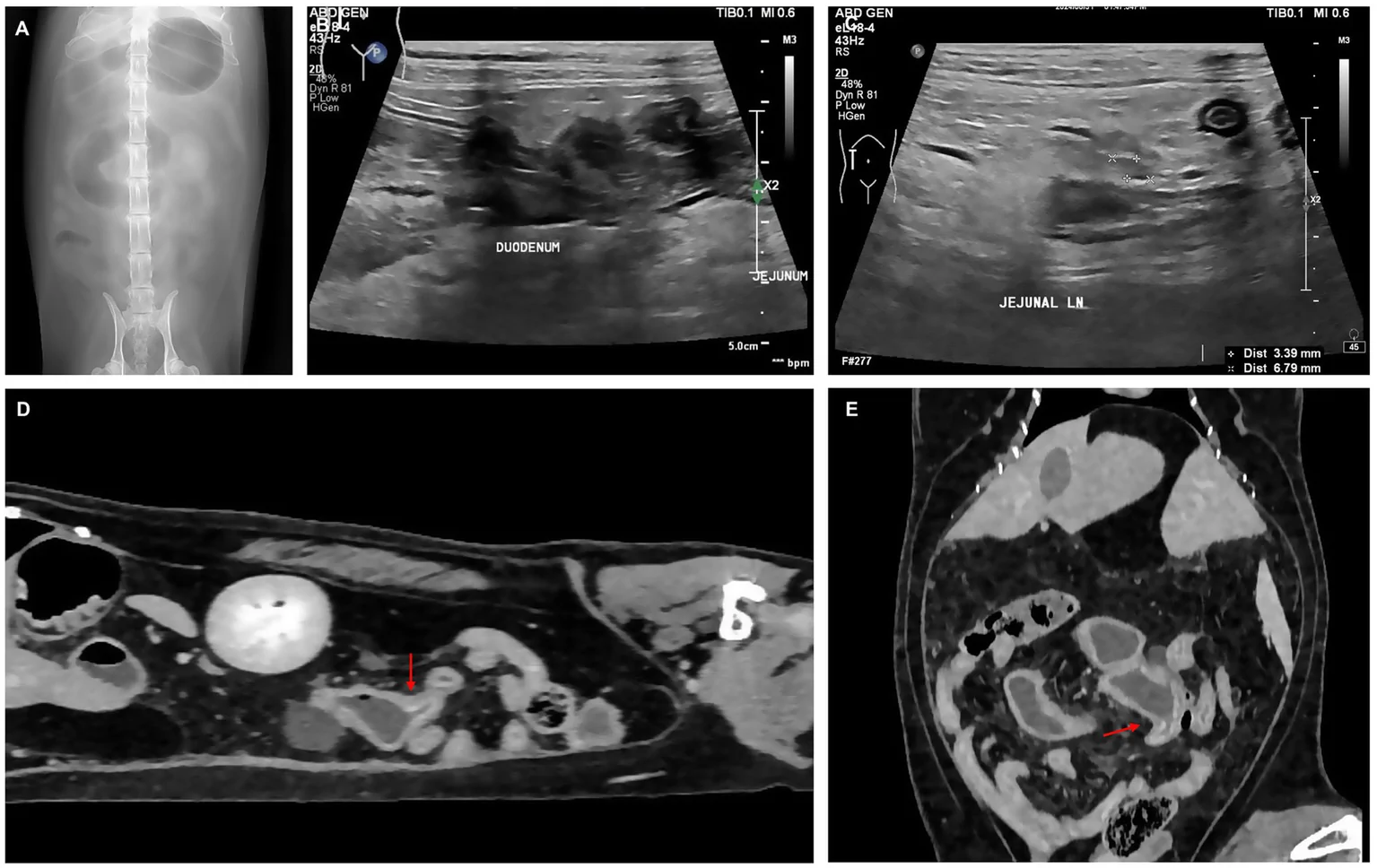
Medical imaging findings of the fibrostenotic lesion causing mechanical small intestinal obstruction in Case 2. **(A)** Ventrodorsal abdominal radiograph showing severe segmental small intestinal dilation. **(B)** Abdominal ultrasonographic image showing an abrupt stenotic lesion with proximal intestinal dilation. **(C)** Ultrasonographic image showing mild enlargement of a jejunal lymph node. **(D,E)** Transverse and dorsal contrast-enhanced computed tomographic images showing the focal jejunal stenotic lesion causing mechanical obstruction; red arrows indicate the stenotic segment.

Additional immunohistochemical staining revealed diffuse cytoplasmic STAT3 immunoreactivity in a moderate proportion of mucosal lymphocytes and diffuse strong cytoplasmic immunoreactivity in enterocytes. In the submucosa, diffuse STAT3 immunoreactivity was observed in macrophages, neutrophils, and fibroblasts (Figures 3B,D). STAT5b immunohistochemistry revealed rare cytoplasmic immunoreactivity in mucosal lymphocytes, diffuse weak cytoplasmic immunoreactivity in enterocytes, and scattered weak cytoplasmic immunoreactivity in submucosal macrophages and neutrophils (Figure 3F). Phospho-JAK1 immunohistochemistry showed predominantly weak cytoplasmic immunoreactivity in mucosal enterocytes, with scattered strong staining in a small proportion of cells (Figure 3H). The semi-quantitative immunohistochemical findings are summarized in Supplementary Table 2.

The owner elected prednisolone monotherapy due to chemotherapy-related adverse effect concerns. The cat remained relatively stable for approximately 1 year after intestinal resection. During follow-up, clinical signs and ultrasonographic intestinal wall thickening worsened during prednisolone tapering and improved after dose escalation. A suspected recurrent stenotic lesion with partial obstruction subsequently developed at the surgical site, although luminal patency was maintained.

After further clinical progression, chlorambucil (2 mg/cat PO every other day) was added. Clinical signs re-stabilized, and prednisolone was gradually tapered. However, weight loss, anorexia, and diffuse small-intestinal wall thickening recurred. Marked progression of the narrowed segment was not observed during the final follow-up examination. Despite supportive care, the cat died 502 days after the intestinal resection.

## 3 Discussion

Unlike previous reports describing intestinal strictures or histopathologic fibrosis, the present two cases illustrate longitudinal clinical and imaging changes associated with fibrostenotic remodeling during the course of feline CE (10–12). In Case 1, diffuse mural abnormalities preceded a focal jejunal stricture; Case 2 showed recurrent mural thickening and postoperative restenosis. Both cats developed fibrostenotic disease in inflammatory and neoplastic CE.

Both cases showed collagen deposition, fibrotic mural remodeling, and luminal narrowing, but the context of fibrosis differed. In Case 1, fibrosis was associated with non-neoplastic lymphoplasmacytic inflammation, submucosal fibroplasia, and mature granulation tissue. In Case 2, it was associated with clonal neoplastic T-cell infiltration and inflammatory and stromal responses. Thus, LPE and LGITL differ in the lymphoid process and fibrosis context but share a fibrostenotic phenotype that is morphologically similar to that described in Crohn’s disease, without implying pathogenic equivalence (8, 9). Therefore, a comparable inflammation-associated fibrotic process may have contributed to stricture formation in these cases (10–12).

STAT3-related signaling may link persistent intestinal inflammation, lymphoma-associated cellular interactions, and fibrostenotic remodeling (13, 16). In humans with fibrostenotic Crohn’s disease, STAT3 signaling in intestinal mesenchymal cells increases TGF-β1 transcription and type I collagen production (16). In these cases, STAT3 immunoreactivity was identified in lymphoid and stromal cells in fibrostenotic lesions. In Case 1, it was strong in lamina propria and submucosal lymphocytes and fibroblasts. In Case 2, it was identified in mucosal lymphocytes, submucosal macrophages, neutrophils, and fibroblasts. Semi-quantitative evaluation further demonstrated variation in the extent and intensity of STAT3 immunoreactivity among the evaluated cell populations in both cases (Supplementary Table 2). These findings suggest a possible association between STAT3-related signaling and inflammatory-stromal interactions in the fibrostenotic lesions of these cases.

Although the observed immunoreactivity does not establish a causal role for STAT3 in fibrosis, the detection of STAT3 immunoreactivity in both fibrostenotic lesions provides a rationale for further investigation of the JAK-STAT3 pathway as a potential therapeutic target in feline fibrostenotic CE. In humans, the selective JAK1 inhibitor upadacitinib is effective for moderate-to-severe Crohn’s disease (17). Direct STAT3 inhibition ameliorated experimental colitis and reduced profibrotic signaling in strictured Crohn’s disease intestine-derived cells (16, 18), supporting investigation of modulation to reduce persistent intestinal inflammation and subsequent fibrostenotic remodeling in cats.

Given JAK-STAT signaling involvement in intestinal inflammation and total STAT3 immunoreactivity in both fibrostenotic lesions, oclacitinib, a veterinary JAK1-preferential inhibitor administered to cats, may be a clinically accessible candidate for drug-repurposing studies on feline CE (19). However, its use is not currently recommended for this condition.

The different postoperative courses in the two cats may also reflect differences in the surgical extent and residual microscopic disease. In Case 1, resection was limited to the grossly stenotic segment, and submucosal fibroplasia with moderate lymphoplasmacytic infiltration extended to the distal edge of the submitted tissue, suggesting microscopic extension beyond the visible lesion. In Case 2, wide-margin resection included an adjacent grossly normal bowel, which may have reduced residual microscopic disease and contributed to prolonged postoperative stability. These findings suggest that the extent of surgical resection might be relevant to postoperative outcome in cats with suspected fibrostenotic enteropathy.

The limitations of this study included the presumptive clinical diagnosis in Case 1, only two cats with different underlying diagnoses, histologic fibrosis distributions, treatments, and surgical approaches, unknown cause of sudden death in Case 1, no postmortem examination, and inability of total STAT3 immunohistochemistry to determine whether STAT3-related signaling contributes to fibrostenotic remodeling or whether the observed immunoreactivity reflects a secondary response to chronic inflammation and tissue remodeling in feline CE. Further studies of STAT3-related signaling pathways in intestinal fibrosis in feline CE are required.

## 4 Conclusion

These cases add new clinical observations to the spectrum of fibrostenosis in cats with CE. The similar fibrostenotic phenotype observed in these two cats suggests that comparable structural remodeling might occur in both inflammatory and neoplastic forms of feline CE. Serial imaging documented progression and suspected postoperative recurrence, while total STAT3 immunoreactivity in both lesions supports investigating JAK-STAT3 signaling in feline intestinal fibrosis related to CE. Additional limitations included the lack of dedicated negative-control sections and objective quantitative image analysis of immunolabeling. Further studies are needed to determine whether modulation of the JAK-STAT3 pathway can alter fibrostenotic progression or postoperative recurrence.

## Data Availability

The original contributions presented in the study are included in the article/Supplementary material, further inquiries can be directed to the corresponding author.
