# Supplementary material for: Fibrostenotic intestinal stenosis associated with chronic enteropathy in two cats: case report
Source: Front Vet Sci. 2026 Oct 12;13:1950860. doi: 10.3389/fvets.2026.1950860 (PMC13698899; doi:10.3389/fvets.2026.1950860)
Supplement: Supplementary file 1 [file Data_Sheet_1.PDF]

Supplementary Table 1. Primary antibodies used for JAK–STAT-related immunohistochemistry.

Target	Catalog no.	Clone	Provider	Final dilution	Positive control	Negative control
Total STAT3	ab68153	EPR787Y	Abcam	1:200	Feline lymph node; diffuse immunoreactivity in activated lymphocytes	Not performed*
Total STAT5b	ab178941	EPR16671	Abcam	1:500	Feline lymph node; diffuse immunoreactivity in activated lymphocytes	Not performed*
Phospho-JAK1	ab138005	EPR1899(2)	Abcam	1:100	Feline lymph node; immunoreactivity in a subset of activated lymphocytes	Not performed*

* Dedicated negative-control sections were not prepared because of limited tissue availability.

Supplementary Table 2. Semi-quantitative evaluation of JAK–STAT-related immunolabeling in intestinal lesions of Cases 1 and 2.

Marker	Case	Tissue compartment	Cell population	Positive cells (%)	Staining intensity
Total STAT3	1	Mucosa	Lamina propria lymphocytes	60	Strong
Total STAT3	1	Mucosa	Enterocytes	100	Moderate
Total STAT3	1	Submucosa	Lymphocytes	80	Strong
Total STAT3	1	Submucosa	Fibroblasts	50	Strong
Total STAT5b	1	Mucosa	Lamina propria lymphocytes	30	Strong
Total STAT5b	1	Mucosa	Enterocytes	100	Weak
Phospho-JAK1	1	Mucosa	Lamina propria lymphocytes	<20	Strong
Phospho-JAK1	1	Mucosa	Enterocytes	80	Predominantly weak; focal strong (<10%)
Total STAT3	2	Mucosa	Mucosal lymphocytes	50	Strong
Total STAT3	2	Mucosa	Enterocytes	100	Strong
Total STAT3	2	Submucosa	Macrophages and neutrophils	60	Moderate
Total STAT3	2	Submucosa	Fibroblasts	80	Moderate
Total STAT5b	2	Mucosa	Mucosal lymphocytes	<20	Weak
Total STAT5b	2	Mucosa	Enterocytes	100	Weak
Phospho-JAK1	2	Mucosa	Enterocytes	80	Predominantly weak; focal strong (<10%)

Immunolabeling was evaluated semi-quantitatively in approximately five representative microscopic fields for each evaluated cell population. The proportion of immunoreactive cells was visually estimated without individual cell counting, and staining intensity was categorized as weak, moderate, or strong.

For phospho-JAK1-positive enterocytes, the predominant staining pattern was weak, with focal strong staining in <10% of enterocytes.
